# Cost-effectiveness analysis of adebrelimab in combination with chemotherapy for first-line treatment of extensive-stage small cell lung cancer

**DOI:** 10.1371/journal.pone.0325171

**Published:** 2025-06-13

**Authors:** Zhiwei Zheng, Huide Zhu, Ling Fang

**Affiliations:** 1 Department of Pharmacy, Cancer Hospital of Shantou University Medical College, Shantou, China; Arsi University College of Health Sciences, ETHIOPIA

## Abstract

**Objective:**

This study aims to assess the cost-effectiveness of adebrelimab in combination with chemotherapy as a first-line treatment for extensive-stage small cell lung cancer (ES-SCLC) from the Chinese medical perspective.

**Methods:**

We have designed a partitioned survival model. The model integrates clinical information pertaining to overall survival, progression-free survival, adverse events and cost data. Our primary measure of outcome in this model is quality-adjusted life years (QALY) and the incremental cost-effectiveness ratio (ICER). The model adopted a willingness to pay (WTP) threshold of $40,343.68 per QALY. To account for uncertainty in the model parameters, we conducted sensitivity analyses.

**Results:**

The total expenditure for the adebrelimab group was $79,549.34, compared to $6,436.80 for the chemotherapy group. The adebrelimab cohort achieved an incremental gain of 1.25 QALY, resulting in an additional cost of $73,112.54. Consequently, the ICER for the adebrelimab group was determined to be $58,490.03 per QALY, surpassing the WTP threshold of $40,343.68 in China. The sensitivity analyses demonstrated the robustness of the findings across various scenarios.

**Conclusion:**

This cost-effectiveness analysis indicates that adebrelimab plus chemotherapy as a first-line treatment for ES-SCLC was not cost-effective in China with a WTP of $40,343.68.Reducing the cost of adebrelimab promises to improve the cost-effectiveness of this treatment regimen.

## 1. Introduction

Small cell lung cancer (SCLC) remains a major challenge in the field of oncology due to its aggressive nature and limited treatment options [1]. Accounting for approximately 15%-20% of all lung cancer cases, SCLC is characterized by its rapid growth, early metastasis, and overall poor prognosis [2]. Despite recent advancements in therapeutic approaches, the management of SCLC continues to be a daunting task. The fast-paced progression of SCLC is a notable characteristic that sets it apart from other lung cancer subtypes. This aggressive nature often leads to the dissemination of cancer cells to distant organs, resulting in early metastasis. Consequently, many patients are diagnosed with extensive-stage SCLC(ES-SCLC), further complicating treatment strategies and significantly reducing the overall survival rates. In fact, the 5-year survival rate for extensive-stage SCLC is dishearteningly low, ranging from a mere 6% to 7% [3].

Recently, there has been a significant breakthrough in the treatment of ES-SCLC with the emergence of immunotherapy as a promising avenue [4].In recent clinical trials, immune checkpoint inhibitors(ICIs) have emerged as a leading class of immunotherapeutic agents. These inhibitors work by blocking the interaction between immune checkpoint proteins, such as programmed cell death protein 1(PD-1) and programmed cell death protein ligand 1(PD-L1) [5]. Currently, the CAPSTONE-1 clinical study has yielded compelling evidence, solidifying adebrelimab as a pioneering PD-L1 inhibitor developed domestically in China [6]. The research findings revealed a median overall survival (OS) of 15.3 months in the group receiving adebrelimab in combination with chemotherapy, which presented an extension of 2.5 months compared to the placebo combination chemotherapy group. Adebrelimab’s efficacy stems from its ability to precisely target the PD-L1 pathway. By selectively blocking the interaction between PD-L1 and its receptor PD-1, adebrelimab effectively restores the immune system’s inherent capacity to combat cancer cells [7]. With marketing approval obtained in March 2023, adebrelimab has emerged as a promising therapeutic addition to the armamentarium of treatment options available for healthcare professionals. Notably, in combination with carboplatin and etoposide, adebrelimab has demonstrated remarkable potential as a first-line therapy specifically tailored for patients diagnosed with ES-SCLC.

However, while the clinical results of this study show promise, it is crucial to consider the cost-effectiveness implications of combining adebrelimab with chemotherapy. This treatment approach incurs additional costs compared to chemotherapy alone. In addition to assessing efficacy, cost-effectiveness is a significant factor in treatment decisions and healthcare policy-making [8].The economic burden of ES-SCLC in China is a significant issue due to the high incidence rates and late-stage diagnosis. Direct medical expenses for ES-SCLC, including hospitalization and chemotherapy, can exceed CNY71,401.92 per year per patient [9]. Additionally, the use of immunotherapy can add an additional cost.Although some immunotherapies are included in China’s National Reimbursement Drug List, coverage remains limited, leading to high co-payments for patients. Furthermore, there are regional disparities in healthcare access, with urban centers more likely to adopt novel therapies compared to resource-constrained regions.These economic and systemic barriers result in delayed or avoided use of immunotherapy in ES-SCLC patients. To address these challenges, cost-effectiveness for high-value ICIs is critical. For instance, a study conducted in the United States aimed to assess the cost-effectiveness of atezolizumab or durvalumab, in combination with etoposide and platinum-based drugs, among patients with extensive small cell lung cancer. The study findings revealed that neither atezolizumab nor durvalumab provided a cost-effectiveness advantage over standard chemotherapy [10].This approach allows healthcare providers and policymakers to optimize resource allocation based on cost-effectiveness assessments and ensure the maximum utilization of resources [11]. Thus, the main objective of this study was to evaluate the cost-effectiveness of adding adebrelimab to chemotherapy versus chemotherapy alone as the first-line treatment for ES-SCLC from the perspective of the Chinese healthcare system.

## 2. Method

### 2.1. Economic evaluation model structure

**Fig 1:**
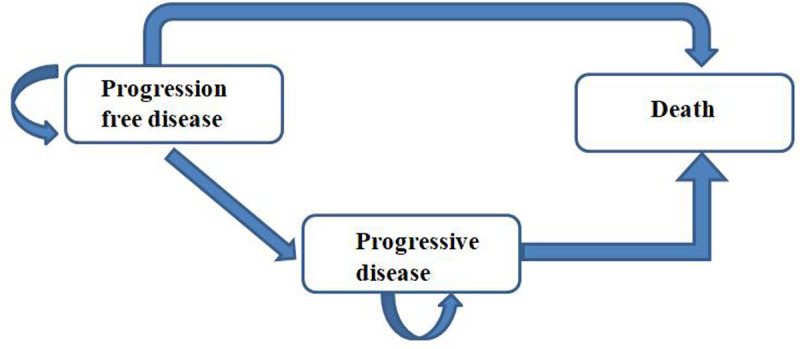
The structure of the model.

We developed a partitioned survival model using TreeAge Pro 2022 software, based on data from the CAPSTONE-1 study. The model incorporates three distinct and mutually exclusive health states: progression-free survival, disease progression, and death. In order to accurately represent the clinical dosing regimen, we defined the model’s cycle period as 3 weeks. Based on the simulation results, it was observed that 99% of patients in the chemotherapy group reached the state of death within a 10-year timeframe. Therefore, the simulation duration for this model was set at 10 years. Additionally, it is assumed that both patient groups start in the progression-free survival state upon entering the model, and each cycle allows them to be in only one health state, receiving the corresponding treatment. The structure of the model is depicted in Fig 1. Statistical analysis and modeling for this study were conducted using TreeAge Pro 2022.

### 2.2. Clinical date and assumptions

Based on the data derived from the CAPSTONE-1 study, the model population was assumed to possess certain characteristics. Firstly, individuals had to be of a minimum age of 18 years. Secondly, their diagnosis of extensive stage small cell lung cancer had to be confirmed through histological or cytological testing. Furthermore, they should not have undergone any systemic therapy for their extensive stage small cell lung cancer. It was also stipulated that participants should have an Eastern Cooperative Oncology Group (ECOG) physical status score of either 0 or 1. Additionally, individuals were required to have a life expectancy of at least 12 weeks.

According to the dosing regimen described in the CAPSTONE-1 study, the chemotherapy-only group was administered intravenous carboplatin at an AUC of 5 mg-min/mL on day 1 of each cycle. Intravenous etoposide was given at a dosage of 100 mg/m^2^ on days 1–3. This treatment cycle was repeated every 3 weeks, up to a maximum of 6 cycles, followed by maintenance therapy with a placebo.

In contrast, the adebrelimab group followed a similar regimen to the control group, with the addition of intravenous adebrelimab at a dose of 20 mg/kg on day 1 of each cycle. The administration of chemotherapeutic agents was limited to 6 cycles every 3 weeks, followed by maintenance therapy with adebrelimab until disease progression occurred.

In instances of disease progression, the current treatment regimen was promptly discontinued in the CAPSTONE-1 study and substituted with second-line therapy. Although the second-line treatment regimen was not explicitly disclosed, the study assumed topotecan based on recommendations from the Chinese Clinical Oncology (CSCO) Guidelines for the Treatment of Small Cell Lung Cancer 2024.

We defined the willingness-to-pay (WTP) threshold at US$40,343.68 per quality-adjusted life year (QALY). This threshold corresponds to three times the gross domestic product (GDP) per capita for the year 2024 [12]. All monetary values relevant to this study were converted from Renminbi (RMB) to United States dollars (USD) using the China central bank official average exchange rate of RMB 7.12 to USD in 2024 [12].

The treatment duration employed in our model was established using data from the CAPSTONE-1 clinical trial. Specifically, the median treatment duration for the adebrelimab group was determined to be 5.6 months, with a range of 4.6 to 6.7 months. In comparison, the chemotherapy group had a median treatment duration of 4.6 months, ranging from 4.3 to 5.5 months.

To ensure the robustness of our study model, we incorporated treatment-related adverse events, taking into account grade 3−4 serious adverse events (SAEs) resulting from the treatment intervention. We included adverse events that had an incidence exceeding 5% in the CAPSTONE-1 clinical trial, as these events were deemed integral components of our model.

### 2.3. Model transfer probability

We employed the GetData Graph Digitizer 2.25 software(https://getdata-graph-digitizer.com/) to extract the probabilities of metastasis for each treatment group from the survival curves obtained in the CAPSTONE-1 trial. In order to accurately determine the distribution that provides the most accurate predictions of patient survival, we generated simulated curves using various distribution options in the R software [13]. These options included the weibull, log-normal,log-logistic,gompertz,exponential and gamma distributions. Subsequently, we utilized two widely accepted criteria, namely the akaike information criterion (AIC) and the bayesian information criterion (BIC), to assess the goodness of fit for each distribution [14]. Additionally, we conducted visual tests to further evaluate the appropriateness of each distribution.The log-logistic model showed lower AIC/BIC values compared to parametric alternatives, indicating superior goodness of fit (S1 Table). Refitting the survival curve with a log-logistic distribution better fits the observed Kaplan-Meier plots in ES-SCLC, particularly when long-term survivors are present (S1 Fig).

During the follow-up period of the trial, the model was executed to gather survival rates directly from the survival curves. The distribution of individuals in each health state was obtained directly from these survival curves. Subsequently, when the model was run after the follow-up period, we employed the best-fitting log-logistic distribution to estimate survival times and determine the survival function, denoted as S(t). The calculation of the survival function relies on the time-transition probability, which follows the expression $S(t=\;1/(1\;+\;{\lambda t}^{\gamma })$ [15]. The survival function S(t) represents the probability that an individual survives beyond a specific time point t and can be derived from the log-logistic distribution. It is characterized by a hazard function that initially rises to a peak and then declines, indicating a higher risk of mortality shortly after diagnosis followed by a stabilization for long-term survivors.The shape parameter γ in the survival function controls the shape of the hazard curve, determining the rate at which the risk of mortality increases and decreases over time. A higher value of γ indicates a more pronounced rise and fall in the hazard, while a lower value results in a less dramatic pattern.On the other hand, the scale parameter λ in the survival function determines the spread of events over time. A larger λ value indicates a wider distribution of survival times, whereas a smaller λ value leads to a more concentrated distribution. The shape parameter γ and the scale parameter λ for the log-logistic distribution were estimated and their values are presented in Table 1.

**Table 1:** Log-logistic distribution survival curve parameters.

Parameters	OS survival curve		PFS survival curve	
	Adebrelimab group	Chemotherapy group	Adebrelimab group	Chemotherapy group
Shape (γ)	1.961	2.676	2.104	5.654
Scale (λ)	0.00428	0.00100	0.0184	0.00119

### 2.4. Costs and utility

In this study, we focused on direct medical costs, which encompassed the costs of adebrelimab and chemotherapy drugs, follow-up care, best supportive care, and management of adverse reactions. Drug cost data were sourced from Yaozhi.com (https://www.yaozh.com/), while costs associated with follow-up care, best supportive care, and management of adverse reactions were extracted from relevant literature [16].Yaozhi.com, a platform specializing in drug pricing data, utilizes a robust data collection process directly sourced from China’s National Healthcare Security Administration (NHSA) and provincial procurement platforms. This methodology ensures the accuracy and reliability of the data, as it aligns with the government-reimbursed drug lists. The incidence rates of adverse reactions were obtained from the CAPSTONE-1 clinical trial. For the purpose of calculating chemotherapy dosages, the study assumed a patient with an average weight of 69.6 kg for male and 59.0 kg for female [17], a creatinine clearance of 70 μmol/L, and a body surface area of 1.72 square meters [16].

Health-related quality of life serves as a critical metric for evaluating the impact of an individual’s health status on daily functioning. Utility values typically range from 0 to 1, where 0 denotes the worst conceivable health state and 1 signifies the optimal health state. However, the CAPSTONE-1 clinical trials encounter significant challenges due to the paucity of specific data on health-related utility values. Consequently, the utility values employed in this study have been derived from the existing literature [16]. It is important to emphasize that these selected utility values will undergo comprehensive sensitivity analysis to rigorously assess their influence on the study outcomes.The costs and utility values are detailed in Table 2.

**Table 2:** The input parameters.

Variable	Baseline value	Range		Distribution	Source
		Minimum	Maximum		
Adebrelimab group SAEs (grade≥3) incidence					
Platelet count decreased	0.38	–	–	Beta	[6]
Anaemia	0.27	–	–	Beta	[6]
White blood cell count decreased	0.46	–	–	Beta	[6]
Neutrophil count decreased	0.76	–	–	Beta	[6]
Chemotherapy group SAEs (grade ≥3) incidence					
Platelet count decreased	0.33	–	–	Beta	[6]
Anaemia	0.28	–	–	Beta	[6]
White blood cell count decreased	0.38	–	–	Beta	[6]
Neutrophil count decreased	0.75	–	–	Beta	[6]
Drug cost (US dollar $)					
Adebrelimab per mg	2.25	1.69	2.81	Gamma	[18]
Etoposide per mg	0.45	0.34	0.56	Gamma	[18]
Carboplatin per mg	0.086	0.06	0.11	Gamma	[18]
Costs of SAEs (grade ≥3) events per cycle ($)					
Platelet count decreased	1054.00	790.50	1317.50	Gamma	[16]
Anaemia	585.88	439.41	732.35	Gamma	[19]
White blood cell count decreased	508.42	381.32	635.53	Gamma	[19]
Neutrophil count decreased	88.42	66.32	110.53	Gamma	[19]
Other					
Best supportive care per cycle	359.52	269.64	449.40	Gamma	[16]
Follow-up cost per cycle	55.60	41.70	69.50	Gamma	[16]
Subsequent treatment	854.05	640.54	1067.56	Gamma	[20]
Routine laboratory examinations per cycle	92.50	69.38	115.63	Gamma	[21]
Abdominal CT per cycle	105.90	79.43	132.38	Gamma	[21]
Utility value					
Progression-free disease	0.67	0.50	0.84	Beta	[22]
Progressive disease	0.47	0.35	0.59	Beta	[22]
Platelet count decreased	−0.19	−0.14	−0.24	Beta	[16]
Anaemia	−0.073	−0.05	−0.09	Beta	[19]
White blood cell count decreased	−0.20	−0.15	−0.25	Beta	[19]
Neutrophil count decreased	−0.20	−0.15	−0.25	Beta	[19]
Body surface area(m^2^)	1.72	1.29	2.15	Beta	[16]
Discount rate	0.05	0.04	0.06	Beta	[23]

### 2.5. Sensitivity analyses

In this study, we performed one-way sensitivity analysis and probabilistic sensitivity analysis. For the one-way sensitivity analysis, we examined the impact of variations in input parameters by allowing them to fluctuate by ±25% of their base values. This approach enabled us to assess the potential influence of these parameters on the incremental cost-effectiveness ratio (ICER). A more narrow range (e.g., ± 10%) may potentially underestimate uncertainty, especially for inputs that exhibit high variability, such as hospitalization costs or rates of adverse events. On the other hand, a wider range (e.g., ± 50%) risks overestimating uncertainty, resulting in less actionable findings for policymakers. Striking a balance, a ± 25% threshold captures moderate uncertainty levels without magnifying extreme scenarios. A similar approach can be observed in studies conducted in the Chinese oncology field, such as those analyzing tislelizumab or durvalumab, where sensitivity analyses often employ ±20–25% ranges [24,25].The results of the one-way sensitivity analysis are illustrated using tornado plots.

For the probabilistic sensitivity analysis (PSA), we employed a Monte Carlo simulation technique comprising 10,000 iterations. In this analysis, input parameters were varied according to predefined distribution functions: costs were modeled using a gamma distribution, while utilities were represented by a beta distribution. The findings from the probabilistic sensitivity analysis are illustrated through a scatter plot.

### 2.6. Ethics approval

No ethical approval was required, as only mathematical modeling was used.

## 3. Result

### 3.1. Cost-effectiveness results

The total cost for the adebrelimab group was $79,549.34, compared to $6,436.80 for the chemotherapy group. The adebrelimab group achieved an incremental gain of 1.25 QALYs, incurring an additional cost of $73,112.54. Consequently, the ICER for the adebrelimab group was calculated to be $58,490.03 per QALY, exceeding the WTP threshold of $40,343.68 in China. This indicates that, at present, the adebrelimab regimen may not be considered cost-effective within the Chinese healthcare system. Detailed results of this analysis are presented in Table 3.

**Table 3:** The base case results.

Parameters	Adebrelimab group	Chemotherapy group
Total cost($)	79,549.34	6,436.80
QALY	1.38	0.13
Incremental cost ($)	73,112.54	—
Incremental QALY	1.25	—
ICER ($/QALY)	58,490.03	—

### 3.2. Sensitivity analyses result

**Fig 2:**
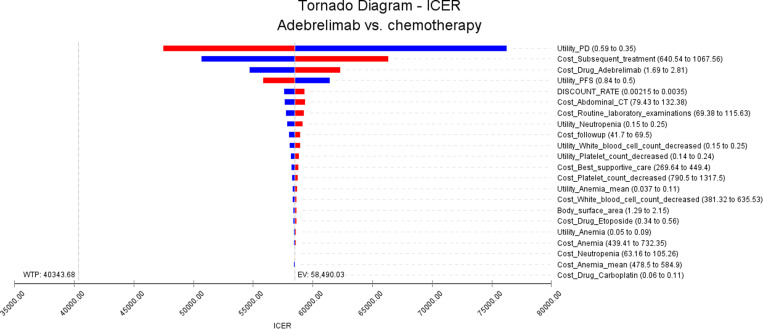
The results of the one-way sensitivity analysis.

The results of the one-way sensitivity analysis are presented in Fig 2. This analysis identified several key factors influencing the ICER. The three most significant factors were found to be the utility value of progressive disease, the cost of subsequent treatment, and the price of adebrelimab. Notably, even after adjusting these input parameters, the resulting ICER values remained above the WTP thresholds. Therefore, the analysis demonstrated that modifications to these parameters within a range of ±25% did not yield substantial changes in the ICER outcomes. These findings align with the conclusions drawn from the base case analysis.

**Fig 3:**
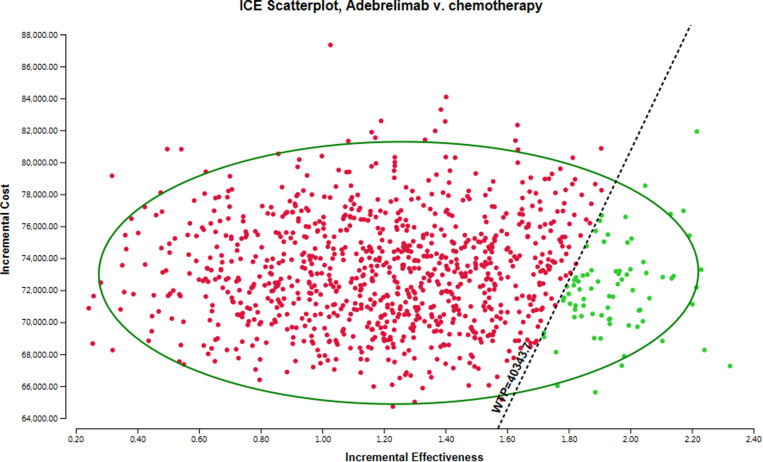
The outcomes of the probabilistic sensitivity analysis.

Fig 3 illustrates the outcomes of the probabilistic sensitivity analysis. Interventions positioned below the linear WTP threshold are classified as cost-effective, signifying that these interventions exhibit more favorable ICER relative to those in other quadrants, thus reflecting lower costs or improved health outcomes. Notably, with a WTP threshold of $40,343.68 per QALY, the adebrelimab group demonstrated only a 7.7% probability of being deemed a cost-effective option compared to the chemotherapy group.

## 4. Discussion

With advancements in drug development, adebrelimab has demonstrated the potential for improved clinical outcomes in patients with ES-SCLC, potentially leading to prolonged survival. As a novel treatment option in the first-line management of ES-SCLC, adebrelimab has garnered significant attention [26,27]. However, a comprehensive economic evaluation is essential to assess its feasibility and economic benefits when selecting appropriate treatment options.

Our study indicates that the combination of adebrelimab and chemotherapy results in an increase of 1.25 QALYs compared to chemotherapy alone; however, this is associated with a substantial incremental cost of $58,490.03. The calculated ICER of $58,490.03 exceeds the WTP threshold in China, which is set at $40,343.68 per QALY. Sensitivity analyses were conducted to evaluate the robustness of our findings, and none of the examined variables significantly affected the results. This suggests that our conclusions are stable and not influenced by underlying assumptions or input parameters. Thus, our analysis clearly demonstrates that, at present, adebrelimab does not represent a cost-effective treatment option compared to chemotherapy alone from the Chinese medical perspective.

It is essential to underscore our opposition to utilizing the findings of pharmacoeconomic analyses, particularly the cost-effectiveness assessment from this study, as a basis for restricting access to adebrelimab. We advocate for the use of these results to inform policy decisions that enhance the overall economic viability of adebrelimab through improvements in the health insurance system, rather than employing current drug utilization strategies to solely reduce costs. The escalating costs of pharmaceuticals have emerged as a significant concern for governments, healthcare systems, and patients [28]. High medication prices not only impose a substantial burden on healthcare budgets but also hinder access to life-saving treatments for those in need [29,30]. Cost-effectiveness analysis serves as a valuable analytical tool that can guide decision-making within the healthcare system, particularly regarding the affordability and value of medications [31]. It is crucial to approach these analyses with the aim of fostering accessibility and ensuring that patients receive the necessary treatments without compromising the sustainability of healthcare resources.

In China, the implementation of a centralized national drug procurement system has emerged as an effective strategy to address the financial strain caused by exorbitant drug prices, particularly for high-value medicines. This approach allows the Chinese government to engage in direct negotiations with pharmaceutical companies, leveraging the country’s considerable purchasing power to attain more favorable pricing terms for critical high-value drugs [32]. In 2016, the government negotiated the prices of three drugs (gefitinib, icotinib, and tenofovir disodoxomil), resulting in a reduction of more than 50% in order to prioritize their reimbursement [33]. To further examine the impact of these negotiations on pricing, a recent study evaluated 103 anti-cancer drugs that were successfully included in China’s medical insurance drug list. The analysis revealed that the median cost of treatment significantly decreased from an average of US $34,460.72 prior to negotiation to US $13,688.79 after negotiation, marking a substantial reduction of up to 60% [34]. Based on our one-way sensitivity analysis, we found that fluctuations in the price of adebrelimab within a 25% range have an impact on the ICER, but this does not change our overall conclusion. However, upon further analysis, we discovered that a significant reduction in the price of adebrelimab, to $1.13 per mg (equivalent to 50% of the current price), would result in an ICER of $39,861.67 per QALY, which is close to the WTP threshold of $40,343.68 per QALY. These results suggest that lowering the price of adebrelimab could potentially make the treatment regimen more economically viable and strengthen its position in cost-effectiveness analysis in China. Furthermore, when we conducted a probabilistic sensitivity analysis with the reduced price of adebrelimab, we found that it had a 48.10% advantage as a regimen option in terms of cost-effectiveness analysis at the WTP threshold of $40,343.68 per QALY. This indicates that, with the reduced price, adebrelimab becomes an even more favorable choice in terms of cost-effectiveness. Lowering the price of adebrelimab could potentially enhance its economic viability and improve its position in cost-effectiveness evaluations in China.

Recently, Long et al. conducted an analysis to evaluate the cost-effectiveness of adebrelimab in combination with chemotherapy compared to chemotherapy alone for patients with ES-SCLC [35]. They concluded that, from the Chinese healthcare system, the combination of adebrelimab and chemotherapy was not economically viable relative to chemotherapy alone.We acknowledge the important findings presented by Long et al.; however, we contend that our study provides unique insights that complement the existing body of evidence. A notable distinction of our research lies in the incorporation of contemporary and updated data. Specifically, the drug pricing utilized in our analysis reflects the most recent updates from 2024, thereby ensuring the accuracy and relevance of our findings. Additionally, the conversion of exchange rates from Chinese RMB to US dollars is based on the latest data available from 2023, which enhances the robustness of our economic evaluation.Moreover, our analysis incorporates updated information regarding China’s GDP for 2023, offering a comprehensive understanding of the economic context pertinent to our study. This further strengthens the validity of our conclusions and provides a more nuanced perspective on the economic implications of treating ES-SCLC in the current healthcare landscape.

The present study has several limitations that should be considered. Firstly, the CAPSTONE-1 study, which formed the basis of our pharmacoeconomic evaluation, had a limited follow-up period. As a result, survival data obtained through the use of parametric survival extrapolation methods may introduce some uncertainty to our findings. Secondly, in real-world clinical practice, the choice of second-line treatment for patients after disease progression can vary according to individual differences. However, in our study, we only assumed topotecan treatment options. This approach may introduce some bias between our results and actual treatment outcomes. Thirdly, our study only included treatment-related serious adverse reactions of grade 3 and above, and did not take into account all adverse reactions. This may introduce some bias in the cost and utility values that we derived, compared to the actual outcomes observed in clinical practice. Nevertheless, the results from the one-way sensitivity analyses suggest that fluctuations in costs associated with grade 3 or higher adverse events did not significantly alter the incremental cost-effectiveness ratio (ICER) values, which consistently remained above the established willingness-to-pay threshold. This stability in ICER values underscores the robustness of our conclusions.

Despite these limitations, our study provides valuable insights into the pharmacoeconomic evaluation of the treatment options for small cell lung cancer. Future studies with longer follow-up periods and consideration of a broader range of treatment options and adverse reactions would help to further improve our understanding in this area.

## 5. Conclusion

Based on the findings of this comprehensive study, the economic evaluation indicates that the combination of adebrelimab with chemotherapy does not represent a cost-effective first-line treatment for ES-SCLC. However, it is proposed that by reducing the price of adebrelimab to $1.13 per mg, the treatment could achieve cost-effectiveness when assessed against the prevailing WTP threshold in China.

## Supporting information

S1 Table Comparison of survival models distribution. (DOCX)

S1 Fig The distribution of survival curve. A: Modes simulation visual overall survival curve of adebrelimab group; B: Modes simulation visual overall survival curve of chemotherapy group; C: Modes simulation visual progression-free survival curve of adebrelimab group; D: Modes simulation visual progression-free survival curve of chemotherapy group.
(JPG)
