# Supplementary material for: Cost-effectiveness analysis of adebrelimab in combination with chemotherapy for first-line treatment of extensive-stage small cell lung cancer
Source: PLoS One. 2025 Jun 13;20(6):e0325171. doi: 10.1371/journal.pone.0325171 (PMC12165369; doi:10.1371/journal.pone.0325171)
Supplement: S1 Table — (DOCX) [file pone.0325171.s001.docx]

**Supplementary Table 1. Comparison of survival models distribution**

|  | AIC | | BIC | |
| --- | --- | --- | --- | --- |
|  | Adebelizumab group | Chemotherap**y** group | Adebelizumab group | Chemotherap**y** group |
| PFS |  |  |  |  |
| Weibull | 1132.192 | 1029.048 | 1139.068 | 1035.941 |
| **Log-logistic** | **1076.896** | **974.110** | **1083.773** | **981.004** |
| Log-normal | 1083.361 | 1009.200 | 1090.237 | 1016.094 |
| Gompertz | 1142.795 | 1091.539 | 1149.671 | 1098.433 |
| Exponential | 1141.757 | 1113.720 | 1145.195 | 1117.167 |
| Gamma | 1119.814 | 1004.957 | 1126.690 | 1011.851 |
| OS |  |  |  |  |
| Weibull | 1186.324 | 1309.252 | 1193.200 | 1316.145 |
| **Log-logistic** | **1176.085** | **1294.855** | **1182.962** | **1304.749** |
| Log-normal | 1176.580 | 1314.487 | 1183.456 | 1321.381 |
| Gompertz | 1202.709 | 1339.590 | 1209.585 | 1346.484 |
| Exponential | 1211.928 | 1386.131 | 1215.366 | 1389.578 |
| Gamma | 1181.476 | 1302.731 | 1188.352 | 1309.624 |

AIC: Akaike information criterion; BIC: Bayesian Information Criterion; OS: Overall survival; PFS: Progression-free survival;
