# Supplementary figures and images for: Cost-effectiveness analysis of adebrelimab in combination with chemotherapy for first-line treatment of extensive-stage small cell lung cancer
Source: PLoS One. 2025 Jun 13;20(6):e0325171. doi: 10.1371/journal.pone.0325171 (PMC12165369; doi:10.1371/journal.pone.0325171)

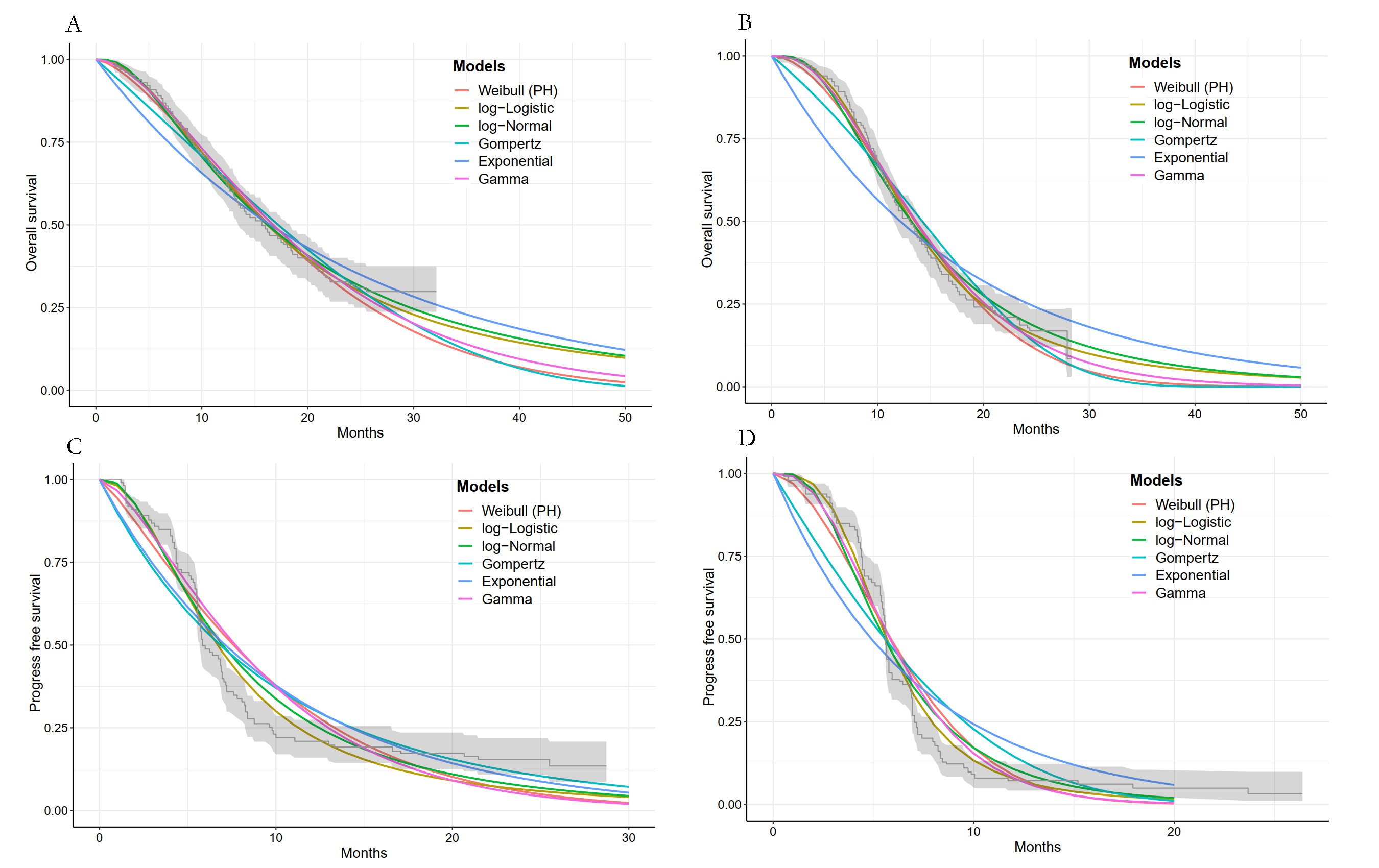

Supplement: S1 Fig — A: Modes simulation visual overall survival curve of adebrelimab group; B: Modes simulation visual overall survival curve of chemotherapy group; C: Modes simulation visual progression-free survival curve of adebrelimab group; D: Modes simulation visual progression-free survival curve of chemotherapy group. (JPG) [file pone.0325171.s002.jpg]
